# The Choice of Medicine as a Career

**Published:** 1915-09-04

**Authors:** 


### The Hospital

The Workers' Newspaper of Administrative Medicine and Institutional Life, Administration, National Insurance and Health.

No. 1525, Vol. LVIII. SATURDAY, SEPTEMBER 4, 1915. PRICE ONE PENNY

### EDUCATIONAL NUMBER.

### THE CHOICE OF MEDICINE AS A CAREER.

Not even the most gloomy of the pessimists anticipates that the present European conflict is likely to continue for another five years, and, as this is the minimum period of the medical curriculum, it follows that the choice of to-day can only realise itself as the commencement of a practical career at the comparatively remote date of 1920. It is on the conditions which are likely to exist at this relatively far-off time that must be based an attempt to appraise what medicine has to offer to those who elect for her service. Long ere that day the conditions of peace will doubtless have re-established themselves, and, though marked by the scars of the struggle, the life of the nation will have resumed its course. There is a necessary obligation to add that for those of military age and physical fitness no need exists to debate with anxiety what medicine might possibly bring them some five years hence. For them is the great opportunity of an immediate and direct contribution to the cause of their country and of human freedom. It is not in the least likely that any of them need to be reminded of this opportunity. They will, we doubt not, be more than ready to postpone any personal ambition for the sake of the large issue which is in all our thoughts. Whether such postponement is long or short, or whether, indeed, the call of the country should mean the supreme sacrifice, the path of duty is plain, and it would indeed be news that Britons refused to follow it.

For those who are free to consider the question we may now briefly discuss some of the issues which must arise when the probable future of medicine has to be contemplated. We will assume that as a profession it commends itself generally to the individual principally concerned. Certainly the career is not one for unwilling recruits, and no one should be urged to it against his will. In a very special degree it calls for deliberate choice, or even for whole-hearted and enthusiastic selection. The man who finds himself embarked in practice without being able to draw from his profession the supreme interest of his life is much to be pitied, and is more than likely to think with bitterness of those who impelled him to so fateful an error. On the other hand, the enthusiastic and deliberate recruit may enter the ranks with confidence, and in the secure conviction that he has a life full of interest and of opportunity in front of him. The great charm of medicine to many is the combination it offers of scientific method in association with human interest, and out of this union those who have the necessary qualities may construct a life rich in educative influences, in mental training, and in the emotional qualities which dignify and discipline manhood. The severity and veracity of science is mingled, and in some sense qualified by a sense of the pathos of human fate, and by some "sight of that immortal sea which brought us hither." Zeal, industry, and even capacity may not gain fortune or wide recognition, but they will make an intellectual home where a man may cherish mental freedom and where gratitude, confidence, and regard will not be wanting. Hence we have none but words of welcome and encouragement for those who are inclined to set their ambitions towards this goal. They may enter a profession which has ancient and great traditions, and may feel sure that they will have opportunities, worthy of their best efforts, both for self-culture and for usefulness to others; and neither is without its reward. All our goodwill is engaged for those who elect for this great experience, and we bid them be of good cheer. Let them remember that it is necessary to cultivate both the courage which dares and also that which endures.

To come to the more special questions of the moment, it seems more than safe to say that the profession of medicine will for many years be able to accommodate large numbers of recruits. Of recent years the entrants have not increased in proportion to the population, and this experience, in association with other circumstances, has meant that the supply of medical practitioners has hardly been equal to the demand. During the last twelve months many young men, who would otherwise have become students of medicine, have joined the Army, and the demand in this direction is increasing rather than diminishing. At the present moment the number of medical students is far below the average level, and hence it is certain that the output of practitioners during the next few years is certain to be a small one. Thus, considered on the most prosaic level, the student who enters the curriculum to-day will, five years hence, find himself in a market where the demand is greater than the supply. His services, to continue the metaphor, will therefore be at a premium, both in the moral and in the financial sense of the term. Already, and by no means entirely as a result of the war, junior members of the profession draw salaries which their fathers a generation ago would have considered to be beyond the dreams of avarice. The circumstances which have created this position are not likely to disappear. Indeed, if anything, they will become more commanding, so that it may be fairly said that the present is a highly fortunate moment at which to embark on the study of medicine. The promise at the end of the curriculum is more than usually attractive in the sense that there need be little or no delay in securing a position to which at least a good living wage is attached. In saying this, we do not forget that before the date here considered we may expect to have once more in civil life many of our colleagues now engaged in military duties. But this will certainly not compensate for the deficient production of doctors during the next four years. The engagement of men with the Forces creates a special urgency at the present moment—an urgency which will in a large measure disappear on their return. An equally acute position is still to appear, when to-day's absence of the medical student will be translated, in the forthcoming years, into a corresponding absence of the medical practitioner. This failure in supply has to be added to the position which existed before the war. Even then the increased number of appointments in the Public Health Department, in school inspection, and in connection with the Insurance Act offered chances to junior practitioners in such abundant measure that the resident posts in hospitals, with their very modest salary, often failed to attract candidates. Economic laws were once, by a great statesman, banished to Saturn. Whether they ever served the sentence may be doubted. In any event they are again in full operation on our planet, and unless we are poorer judges of the future than we believe ourselves to be, the student of to-day will find that on the day, five years hence, that he obtains his diploma the influence of the rule of supply and demand will be strongly and definitely in his favour.

If medical practice can anticipate an improved financial future, it can anticipate also, we think, a more decided scientific future. One of the most influential teachers of the day has written of the present as a time in which "science is flooding everywhere into medicine." The phrase, at least when detached from its context, may perhaps seem to imply an unjust censure on the past. But, however this may be, it is beyond dispute that in a definitely appreciable measure medical opinion and practice are finding an increasingly secure basis in the possession of scientific knowledge. This result arises mainly from two influences. First, the growth of bacteriology and of allied studies has stimulated enormously the search for the causes of disease, and it is manifest that when causes are known a large step has been taken in the search for rational treatment, and, still more, in the direction of anticipation and prevention. In the second place, there are in process of development in medicine methods which carry that distinguishing mark of science, namely, a capacity for measurement. Each of these two influences has some gains already to its credit, and without doubt each will have many more. It is certain, therefore, that a training in scientific habits and methods will not only be emphasised for the student, but also that it will be continued as an influence in the life of the practitioner. Medical education, at least when cultivated under University influences, has always insisted on the importance of the preliminary and scientific portion of the curriculum, though the natural impatience of the student has often seen in these subjects mere hindrances to his active operative career. Physics, chemistry, biology, bacteriology, do at first sight seem remote from the amputation of limbs, but it is certain that they are in medicine ever-increasing forces. Some of their enthusiastic exponents appear to think that they are everything—that patients, like test tubes, can all be numbered and injected. This is an outcome of some measure of success in association with a badly judged perspective. All the same, the student will be well advised to see that his scientific studies are pursued with unqualified thoroughness, for it is the knowledge thus gained, plus the carefully gathered experience at the bedside and in the hospital ward, which the future will expect from the practitioner of medicine. A realisation of this growing influence of science in medical life and work cannot fail to prove to many minds an added attraction to the selection of medicine as a life career.

One other modern tendency may be briefly noted. It is the movement towards organisation and co-ordination. The private medical practitioner is not in the least likely to disappear, more especially as he will to a larger and larger extent be equal to modern developments. Indeed, his general experience and his ability to see medicine "steadily and to see it whole" make him a necessity of the situation, and, for our own part, we expect for him not less but more. The attractions of private practice do not appeal to all minds, and here is where the increase in the organised services of medicine in relation to the community may bring to her ranks some who would otherwise select another career. Developments of this order are prominent and are likely to become still more prominent, so that gifts of organisation and of method and system may reasonably look for a chance of development. The field, indeed, is wide and does not exclude any of the talents so long as these are associated with industry, sincerity, a love of accuracy, and mental honesty. In a final word we will quote—and the truth still abides—what Lord Bacon wrote of a life devoted to medicine, "If men will intend to observe, they shall find much worthy to observe."

